# A NACHT domain-containing protein Ncp is required for appendage-associated unconventional protein secretion in the fungus *Penicillium herquei*

**DOI:** 10.1016/j.isci.2026.116464

**Published:** 2026-06-17

**Authors:** Luwen Yan, Wei Deng, Penglei Qiu, Tongyao Liu, Kaiwen Deng, Liao Zhang, Xiaojing Liu, Jie Fan, Penglin Wei, Dongsheng Wei, Xingzhong Liu

**Affiliations:** 1 State Key Laboratory of Medicinal Chemical Biology, Key Laboratory of Molecular Microbiology and Technology of the Ministry of Education, Department of Microbiology, Frontiers Science Center for Cell Responses, College of Life Science, Nankai University, Tianjin 300071, China

**Keywords:** Molecular biology, Mycology, Cell biology

## Abstract

Unconventional protein secretion (UcPS) enables leaderless proteins to bypass the ER-Golgi pathway, yet its regulation in fungi is not fully characterized. Here, we identify an appendage-associated secretion route in the symbiotic fungus *Penicillium herquei* Ph506 that mediates the extracellular accumulation of leaderless proteins. Functional analyses reveal that Ncp, a highly upregulated NACHT domain-containing protein, is dispensable for appendage formation but required for the efficient secretion of leaderless proteins into these specialized structures under tested conditions. Notably, Ncp depletion suppresses programmed cell death (PCD) features and reduces ionic stress tolerance, suggesting a potential link between NACHT-mediated processes and stress-associated cellular states. Together, this work provides evidence of a specific regulatory mechanism for protein secretion in symbiotic fungi, offering insights into how PCD-related pathways and UcPS may be co-regulated to maintain cellular homeostasis.

## Highlights

• *Penicillium herquei* exports leaderless proteins via unconventional protein secretion
• Ncp contributes to the efficiency of UcPS
• Ncp modulates PCD and is required for ionic stress tolerance
• Ncp coordinates UcPS with cellular stress adaptations and homeostasis

## Graphical abstract

**Figure:**
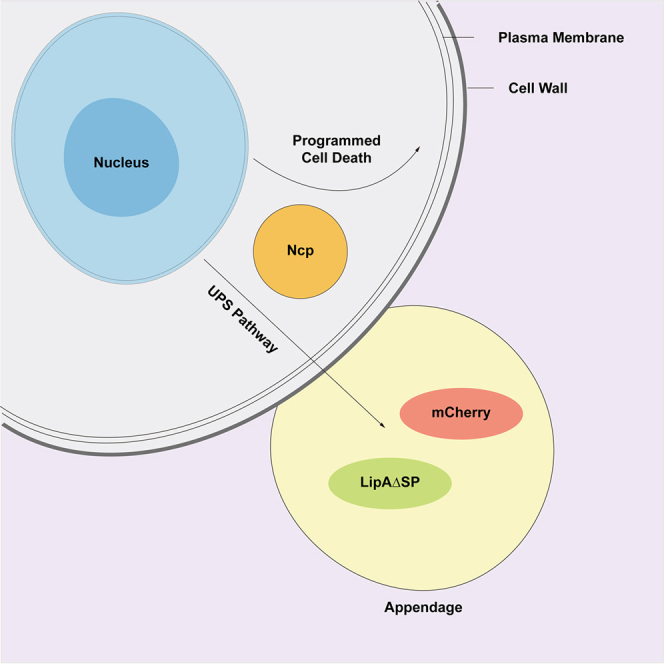

## Introduction

Protein secretion represents a core biological process in eukaryotes that underlies environmental adaptation, intercellular communication, and host interactions.^1^ In the canonical secretory pathway, signal peptide-bearing proteins are co-translationally targeted into the endoplasmic reticulum (ER) lumen, modified in the Golgi apparatus, and subsequently released to the extracellular milieu via vesicular trafficking.^1^^,^^2^ This ER-Golgi-dependent route has long been considered the major pathway of protein export in eukaryotes and is broadly employed by fungi to secrete enzymes, toxins, and effectors.^3^^,^^4^^,^^5^ However, with the advancement of secretomics and extracellular proteomics, numerous extracellular proteins lacking signal peptides have been repeatedly identified across diverse eukaryotic systems, and their export cannot be explained by the classical model.^6^ These findings led to the concept of unconventional protein secretion (UcPS),^6^^,^^7^ which currently encompasses extracellular vesicle-mediated release, direct translocation across the plasma membrane, pore formation, and autophagy-associated pathways.^6^^,^^8^

In mammals and yeast, UcPS has been implicated in inflammation, immune responses, and cellular stress pathways.^9^^,^^10^ To date, some leaderless proteins have been mechanistically characterized in yeasts, such as the Acyl-CoA-binding protein (Acb1p) from *Pichia pastoris*, whose export depends on GRASP, peroxisomes, and autophagosome formation,^11^ and the superoxide dismutase 1 (SOD1) from *Saccharomyces cerevisiae*, whose secretion involves Grh1 and ESCRT factors.^12^ Other leaderless proteins have been detected in extracellular vesicles or extracellular matrices, including enolase from *Saccharomyces cerevisiae* and *Candida albicans*,^13^^,^^14^ as well as the leaderless nigerolysins A from *Aspergillus* spp.^15^ Yet the export mechanisms, evolutionary context, and physiological relevance of these proteins are not yet fully understood,^16^ thereby limiting our understanding of the diversity and ecological strategies of fungal secretory systems.

The leaf-rolling weevil *Euops chinensis* and its symbiotic fungus *Penicillium herquei* Ph506 (hereafter Ph506) form a highly specialized mutualistic association characterized by pronounced structural and functional differentiation that emerged through long-term coevolution.^17^ Morphological examination revealed that matured hyphae of Ph506 develop numerous extracellular structures, termed “appendages”, which are distinct from classical extracellular vesicles or cell wall protrusions.^18^ Specifically, these appendages are defined as spatially discrete, membrane-associated extracellular compartments that lack a chitinous cell wall and remain stably localized on the hyphal surface. Such structures likely represent a specialized functional adaptation emerged through the long-term coevolution between Ph506 and its insect host, serving as dedicated reservoirs for secreted proteins. Proteomic analyses suggest an enrichment of protein components within this appendage, implying potential roles in material exchange or environmental adaptation within the symbiosis. Notably, when mCherry lacking a signal peptide was expressed in Ph506, fluorescence accumulated within appendages rather than remaining intracellular. This observation supports the presence of a noncanonical secretion mechanism capable of exporting leaderless proteins, thus providing a valuable experimental system for dissecting UcPS in filamentous fungi.

To elucidate the molecular basis of this UcPS phenomenon, we integrated temporally resolved transcriptomics during active protein secretion with comparative genomics to identify putative regulators. These analyses revealed that genes encoding NACHT domain-containing proteins are significantly expanded in Ph506 compared to closely related *Penicillium* species, with several candidates showing persistent upregulation synchronized with the secretory phase. The NACHT domain belongs to the STAND ATPase superfamily and was first characterized in animal NOD-like receptors, where it participates in inflammasome assembly and programmed cell death (PCD) regulation.^19^^,^^20^ PCD forms a fundamental component of the fungal life cycle, occurring during sexual and asexual development, aging, and plant-pathogenic interactions, and contributes to stress responses, tolerance, and physiological regulation.^21^ In fungi, the NACHT proteins frequently coexist with LRR or WD40 domains and are considered core factors of heterokaryon incompatibility and PCD pathways.^22^^,^^23^^,^^24^ However, potential links between NACHT domains and fungal membrane dynamics, extracellular structure biogenesis, or protein secretion have not been established.

Fungal secretory systems play central roles in environmental adaptation, host interactions, and symbiosis, often through the secretion of abundant proteins that modulate host immunity or nutrient exchange,^25^ and industrial platform for protein production.^26^ Yet how UcPS in Ph506 is regulated and whether NACHT domain-mediated processes, such as PCD-related pathways, are coordinated with UcPS is not fully characterized. Against this backdrop, we validated the selective export of leaderless proteins in Ph506, and identified a NACHT domain-containing protein (Ncp) as a key regulator of UcPS through genetic perturbation, secretion assays, ion stress responses, and PCD signaling. Together, these findings provide a conceptual framework for the molecular regulation of UcPS in eukaryotes and offer further insight into the evolution and physiology of secretory strategies in symbiotic fungi.

## Results

### Ph506 employs an unconventional pathway for protein secretion

**Figure 1:**
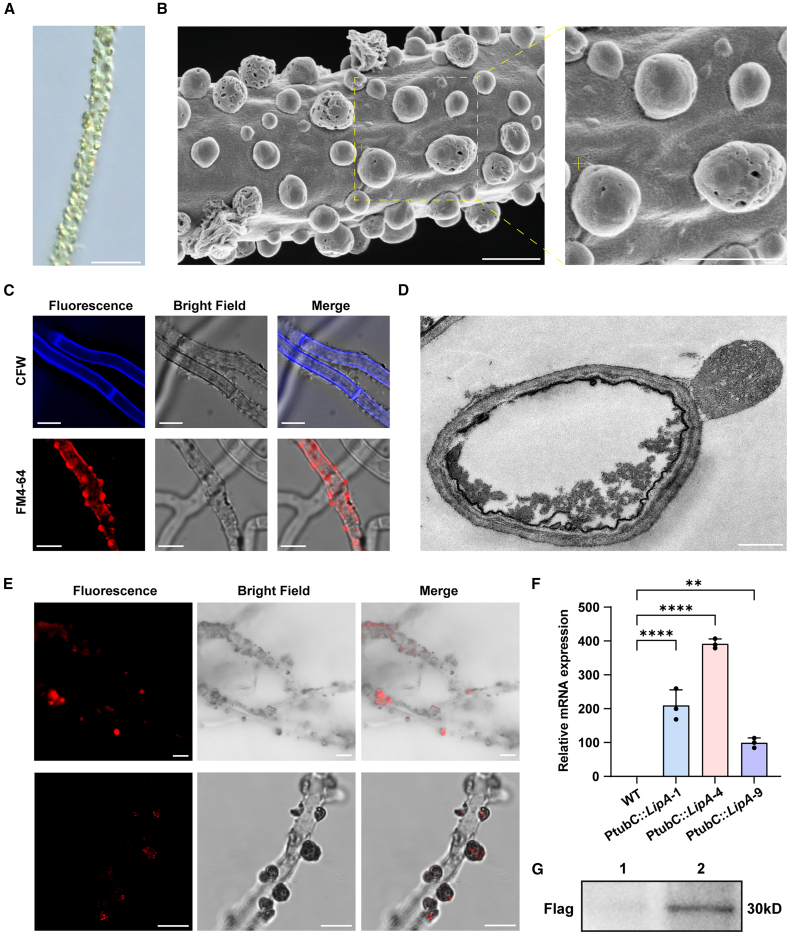
Visualization and validation of unconventional protein secretion in *Penicillium herquei* Ph506 (A) Differential interference contrast (DIC) microscopy (100×) showing Ph506 hyphae densely covered with extracellular appendages. Scale bars, 10 μm. (B) Scanning electron microscopy (SEM) image of Ph506 hyphae bearing abundant surface appendages. Scale bars, 1 μm. (C) Calcofluor White (CFW) and FM4-64 staining of Ph506 hyphae and appendages. Scale bars, 5 μm. (D) Transmission electron microscopy (TEM) of Ph506 hyphal and appendages. Scale bars, 0.5 μm. (E) Fluorescence microscopy of Ph506 expressing cytosolic mCherry, illustrating fluorescence distribution in hyphae. Scale bars, 10 μm. (F) Quantitative real-time PCR analysis of *LipA* transcript levels in transformants expressing signal peptide-less LipA. Data are presented as mean ± SD from *n* = 3 biological replicates. Statistical significance was determined by one-way ANOVA followed by Tukey’s multiple comparisons test; ∗∗*p* < 0.01, ∗∗∗∗*p* < 0.0001. (G) Western blot analysis of appendage-associated proteins detecting LipA expression. Lane 1, proteins extracted from WT appendages; lane 2, proteins extracted from appendages of the P*tubC*::*LipA*-4 strain. See also Figure S1 and Table S1.

To verify the presence of hyphal appendages in Ph506, we observed densely distributed yellowish nodular protrusions on the surface of cultured hyphae by light microscopy and scanning electron microscopy (SEM) (Figures 1A and 1B). These observations suggest that these structures are intrinsic fungal-associated extracellular features rather than non-specific adsorption or medium-derived debris. To further characterize their structural nature, we performed Calcofluor White (CFW) staining; the absence of detectable cell wall-associated fluorescence indicated that these protrusions are not continuous extensions of the fungal cell wall (Figure 1C). Consistently, transmission electron microscopy (TEM) of hyphal cross-sections showed that these structures are located external to an intact and well-defined cell wall boundary, with no disruption of the underlying hyphal architecture (Figure 1D). In addition, FM4-64 staining indicated that these structures are associated with membrane components (Figure 1C). Together, these results reveal that the protrusions represent extracellular, membrane-associated compartments that are physically distinct from the hyphal cell body. We termed these extracellular structures “appendages”. Notably, these appendages represent stable, localized functional domains—potentially evolved through long-term co-evolution within the *P. herquei-E. chinensis* symbiosis—that are distinct from transient secretory vesicles or generalized cell debris (P.Q., J. Liu, L.Y., Y. Huang, H. Xiao, J. Ma, M. Xiang, D.W., X.L., unpublished data). Unlike hyphal tips or simple cell wall outgrowths, appendages lack a rigid chitinous wall and appear to function as dedicated reservoirs for the concentrated sequestration of symbiotic proteins.

When a cytosolic red fluorescent protein, mCherry, was expressed in Ph506, we observed strong fluorescence signals specifically localized to the appendages (Figure 1E). Notably, mCherry lacks a signal peptide (Figure S1A), and its fluorescence was confined to the appendage-associated structures rather than being diffusely distributed in the surrounding medium, suggesting that the extracellular accumulation is unlikely to result from generalized cell lysis. This assay indicates that leaderless proteins can be exported from the cytosol and specifically accumulate within these extracellular compartments. Given that canonical secretion in filamentous fungi relies on signal peptide-dependent targeting through the ER and Golgi apparatus,^27^ the extracellular localization of mCherry is inconsistent with conventional secretion mechanisms and instead supports an active UcPS-like process.

To further substantiate the ability of Ph506 to export leaderless proteins, we engineered a signal peptide-deficient version of the classical secretory enzyme lipase A (LipA, NCBI Protein ID: ABK69591.1) by removing its N-terminal 17-amino-acid signal peptide and introducing an FLAG tag (Figure S1B). Using *Agrobacterium tumefaciens*-mediated transformation, we obtained a high-expression transformant P*tubC*::*LipA*-4 (Figure 1F). Appendage-associated extracellular material was isolated from large-scale cultures, and immunoblot analysis detected LipA in the appendage-associated protein fraction (Figure 1G). Consistent with signal peptide predictions (Figure S1), both mCherry and LipA lack N-terminal secretion signals and therefore cannot enter the canonical ER-Golgi pathway. Taken together, these findings provide evidence for a secretion route that enables leaderless proteins to reach appendage-associated extracellular structures, a process distinct from the canonical secretion pathway.

### Temporal transcriptomic profiling reveals regulatory programs associated with protein secretion

**Figure 2:**
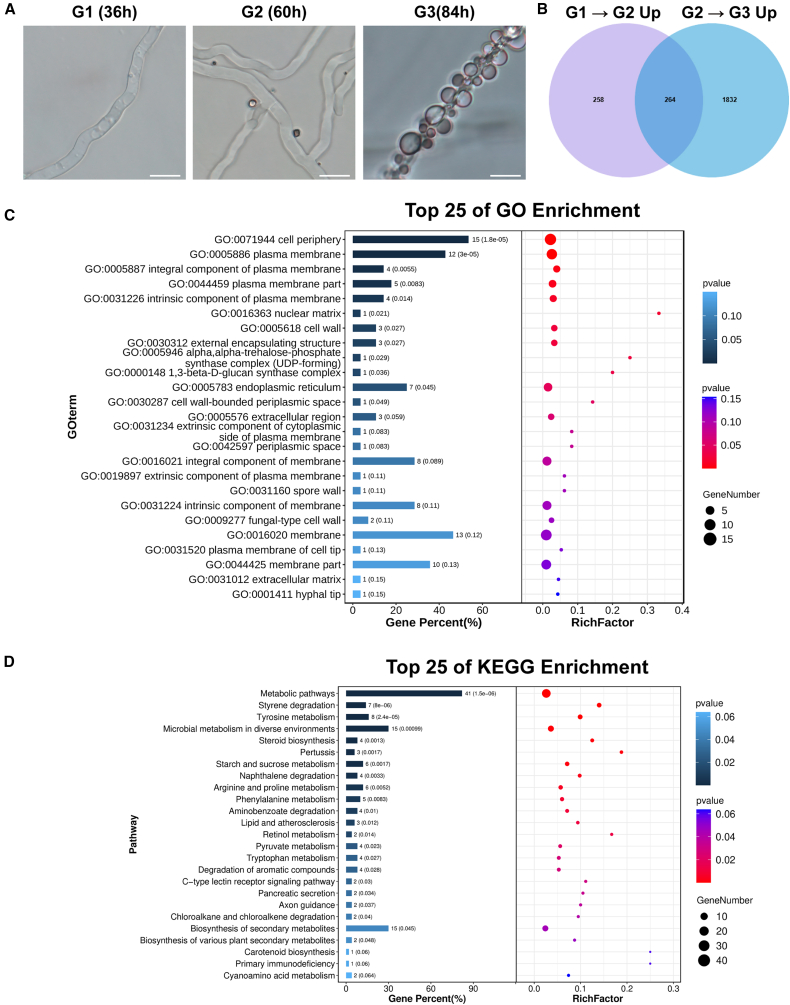
Time-resolved transcriptomic profiling reveals candidate genes associated with appendage-related protein secretion (A) Representative morphology of hyphae at G1 (36 h), G2 (60 h), and G3 (84 h) sampling stages used for time course transcriptomic analysis. Scale bars, 10 μm. (B) Venn diagram showing genes upregulated from G1 to G2 and from G2 to G3, with the intersection representing 264 genes continuously upregulated across all stages. (C) Gene ontology (GO) enrichment analysis of the 264 genes showing sustained upregulation across G1–G3. (D) KEGG pathway enrichment analysis of the same set of 264 continuously upregulated genes. See also Figure S2.

To identify molecular pathways associated with the secretion of leaderless proteins, we focused on genes exhibiting sustained transcriptional upregulation during the transition to an active extracellular protein export state. Based on phenotypic observations, hyphae at 36 h post-inoculation (G1) were well developed but possessed minimal appendages. In contrast, at 60 h (G2) and 84 h (G3), appendage density progressively increased (Figure 2A). We therefore utilized appendage abundance as a morphological proxy for secretory activity, with the G1-G3 transition reflecting a gradual escalation in appendage-associated protein output. Accordingly, these three stages were selected for temporal transcriptomic analysis. Principal-component analysis (PCA) of transcript levels from temporal transcriptomic data across the three stages (36, 60, and 84 h; three biological replicates each) showed clear separation among the stages with strong reproducibility between biological replicates (Figure S2), validating the sampling strategy.

Differential expression analysis identified 522 genes upregulated from G1 to G2 and 2,096 genes upregulated from G2 to G3. Intersection analysis yielded 264 genes that were consistently upregulated across all three stages (Figure 2B), representing a core transcriptional program associated with sustained protein output. To elucidate the functional characteristics of this gene set, gene ontology (GO) and KEGG pathway enrichment analyses were performed.

GO enrichment analysis revealed strong overrepresentation of terms related to the cell periphery, plasma membrane, extracellular region, and hyphal tip-associated compartments, as well as membrane-integrated and membrane-associated components. These categories are consistent with active processes occurring at membrane interfaces during protein translocation. Notably, terms associated with the ER also exhibited significant enrichment. This suggests a coordinated interplay between UcPS and the classical endomembrane system, rather than these pathways operating in complete isolation (Figure 2C).

KEGG pathway analysis further highlighted metabolic and signaling pathways linked to lipid remodeling, carbohydrate metabolism, and environmental adaptation (Figure 2D). Several enriched pathways are associated with stress-responsive signaling and cellular homeostasis, processes known to influence secretion capacity and cell fate decisions.

Collectively, these enrichment patterns do not point to individual secreted cargos but instead converge on biological processes associated with membrane dynamics, metabolic state, and stress signaling. This observation aligns with the established principle that UcPS is a highly regulated process often coordinated with cellular stress responses and membrane trafficking pathways, rather than a passive release of intracellular contents.^16^^,^^28^^,^^29^ These results indicate that the observed transcriptional changes reflect activation of higher-order regulatory programs. Guided by this inference, we systematically examined gene families with potential regulatory roles, which led to the identification of NACHT domain-containing proteins—known regulators of cell fate and stress signaling—as prominent candidates associated with this process.

### NACHT domain-containing proteins involve in the UcPS of Ph506

Subsequently, we performed domain annotation and functional prioritization of the 264 consistently upregulated genes, focusing on candidates potentially involved in stress signal integration. Notably, a subset of these genes encoded proteins containing a NACHT domain. A member of the STAND family of P loop NTPases known to undergo ATP/ADP binding and hydrolysis-driven conformational changes.^19^ In diverse eukaryotes, NACHT proteins function as molecular switches that coordinate stress responses and PCD, linking environmental cues to cellular decision-making.^30^

**Figure 3:**
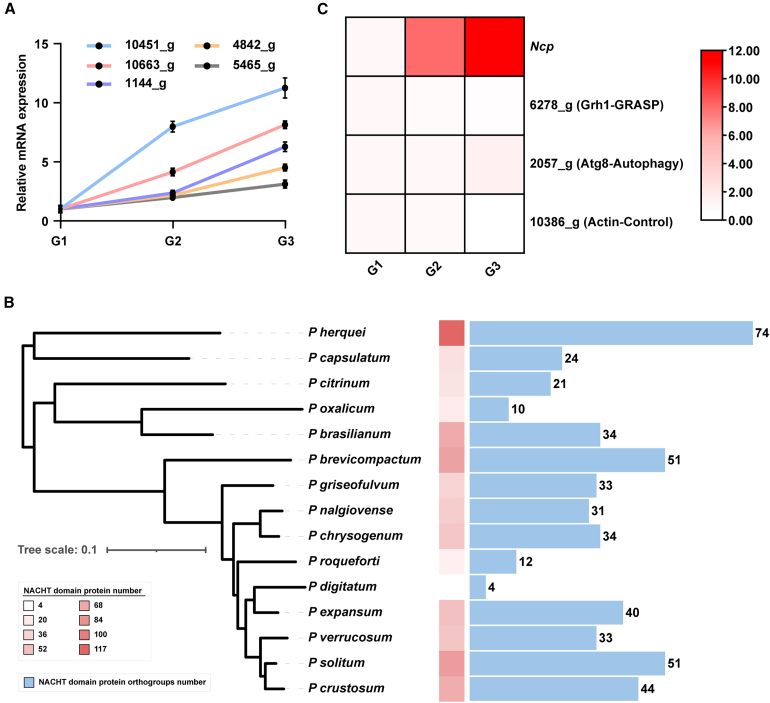
NACHT domain-containing proteins are enriched and transcriptionally activated during appendage-associated protein secretion (A) qPCR validation of NACHT domain-containing gene expression at G1, G2, and G3 stages. Data are presented as mean ± SD from *n* = 3 biological replicates. All target genes exhibit a sustained upregulation profile synchronized with the fungal developmental stages. (B) Comparative enrichment analysis of NACHT domain-containing proteins in Ph506 and 14 related *Penicillium* species. Red squares indicate the number of NACHT domain-containing proteins in each species, with color intensity reflecting abundance. Blue bars represent the number of NACHT-associated orthogroups (as defined by orthology clustering analysis) in each genome. (C) Comparative transcriptional dynamics of *Ncp* and canonical UcPS-related genes. Relative expression levels of *Ncp* and homologs of known unconventional protein secretion (UcPS) regulators were analyzed across three developmental stages (G1, G2, and G3) using time course RNA-seq data. All expression values were normalized to the G1 stage (G1 stage set as 1.0). The heatmap illustrates a robust, 11.26-fold upregulation of *Ncp* synchronized with the peak secretion phase. In contrast, identified homologs, including 6278_g (Grh1) and 2057_g (Atg8), exhibit stagnant expression profiles with no biologically significant fluctuations (|log_2_FC| < 1.0). Actin serves as an internal control for stable baseline expression. See also Tables S1 and S2.

Among the consistently upregulated genes, we identified five encoding NACHT domain-containing proteins (10451_g, 10663_g, 1144_g, 4842_g, and 5464_g). While all five candidates exhibited upregulation in the transcriptomic dataset, quantitative PCR (qPCR) analysis confirmed that 10451_g (designated as *Ncp*) displayed the most robust and significant upregulation across the three sampling points (Figure 3A). This strong transcriptional response, synchronized with the peak secretion phase, suggests that Ncp serves as a key regulator node.

To explore the genomic features associated with the UcPS phenotype, we performed comparative genomic analyses between Ph506 and 14 representative *Penicillium* species. An unrooted phylogenetic tree resolved Ph506 as a distinct clade within the *Penicillium* lineage (Figure 3B). Quantitative comparison revealed a pronounced enrichment of NACHT domain-containing proteins in Ph506 relative to other *Penicillium* species; at the orthogroup level, Ph506 also exhibited an increased number of NACHT-associated orthogroups compared with other species, indicating an expansion of this gene family within its genome (Figure 3B; Table S2). While this enrichment represents a notable feature of the Ph506 genome potentially linked to its symbiotic lifestyle, whether this is a conserved hallmark across other symbiotic fungi remains to be determined given the current taxon sampling.

Critically, we conducted a systematic survey of established UcPS machineries to determine whether Ncp potentially operates through or coordinates with known UcPS core factors within the Ph506 genome. Using protein sequences from *Aspergillus nidulans* and *S. cerevisiae* as queries for BLASTP analysis, we found that the canonical components of several major eukaryotic protein export systems are entirely absent or lack identifiable homologs in Ph506 (Table 1). These include canonical UcPS machineries such as the ESCRT machinery (I, II, and III),^29^ pore-forming GSDMD^31^^,^^32^ the cargo receptor TMED10,^33^^,^^34^^,^^35^ and the deubiquitinase USP19.^36^^,^^37^^,^^38^ Furthermore, while homologs of Atg8^9^^,^^39^ and Grh1^12^^,^^40^ exist in Ph506, their transcriptional levels remained stagnant throughout the peak secretion phase (|log_2_FC| < 1.0), in sharp contrast to the 11-fold upregulation of *Ncp* (Figure 3C). To further eliminate the possibility that highly divergent fungal homologs were missed by direct sequence alignment, we conducted a more sensitive, domain-based search using profile hidden Markov Models (HMMs). This targeted survey focused on fungal-specific gasdermin-like proteins characterized by the HeLo domain (PF14479)^41^ and the TMED/p24 family defined by the emp24 domain (PF01105).^42^^,^^43^ Although these HMM-based searches identified several candidates, an integrated analysis of their genomic localization, membrane topology, and transcriptomic profiles did not reveal a direct functional coupling with *Ncp* (Table 2). Specifically, GSDM-like candidates lacked transmembrane domains, and TMED homologs showed no transcriptional response during peak secretion. While these results suggest that Ncp does not rely on identifiable canonical executors, we cannot exclude the possibility of Ncp acting as an upstream signal activator that converges with highly divergent or post-translationally regulated pore-forming proteins. Together, these findings suggest that Ncp serves as a potential modulator for protein secretion in this symbiotic fungus, potentially representing a specific regulatory mechanism or an upstream activator of uncharacterized downstream executors.

**Table 1:** Genomic survey of canonical UcPS components in the *Penicillium herquei* Ph506 genome

Category	Query Sequence	Query Source	Best Hit ID	Identity	E-value	Status
GRASP55	Grh1	*S. cerevisiae*	6278_g	26.946%	4.23e-26	identified
TMED10	Emp24	*A. nidulans*	none	–	–	not found
GSDMD	Gsd1	*A. nidulans*	none	–	–	not found
ESCRT-I	Vps23	*A. nidulans*	none	–	–	not found
ESCRT-II	Vps22	*A. nidulans*	none	–	–	not found
ESCRT-III	Snf7	*A. nidulans*	none	–	–	not found
Autophagy	Atg8	*A. nidulans*	2057_g	60.345%	3.56e-49	identified
USP19	Ups19	*A. nidulans*	none	–	–	not found
Positive Control	Actin	*A. nidulans*	10386_g	81.818%	0.0	identified

A local BLASTP search (E-value < 1e-5) was conducted using reference sequences from *Aspergillus nidulans*, *Saccharomyces cerevisiae*. “Not Found” indicates no homologous sequences were identified within the Ph506 genome. Actin was used as a positive control to validate search sensitivity.

**Table 2:** Comparative analysis of GSDM-like and TMED homologs in *Penicillium herquei* Ph506 regarding genomic localization, domain annotation, and expression profiles

Candidate Pathway	Gene ID	Scaffold	Domain annotation (Pfam/KEGG/GO)	Expression Pattern (G3 vs. G1)	Predicted TMHs (TMHMM)	Functional Implication
Master Regulator	10451_g (*Ncp*)	Segkk100	NACHT (PF05729)	upregulated	0	master regulator of UcPS
Gasdermin-like	2775_g	Segkk47	HeLo (PF14479) Ankyrin repeat (K21440)	upregulated	0	scaffolding/non-pore-forming
	7108_g	Segkk78	HeLo (PF14479) Protein kinase (GO:0004672)	downregulated	0	signaling/non-pore-forming
TMED	9075_g	Segkk84	Emp24/gp25 L (PF01105)	no change	1	functional receptor; unrelated to *Ncp*
	8753_g	Segkk83	Emp24/gp25 L (PF01105)	downregulated	0	non-functional/cytosolic homolog
	8941_g	Segkk84	Emp24/gp25 L (PF01105)	no change	2	functional receptor; unrelated to *Ncp*
	6916_g	Segkk77	Emp24/gp25 L (PF01105)	downregulated	1	functional receptor; unrelated to *Ncp*

*Ncp* is located on Scaffold Segkk100, while all other candidates are distributed across different scaffolds, indicating a lack of genomic linkage.

### Ncp contributes to the efficient secretion of leaderless proteins

**Figure 4:**
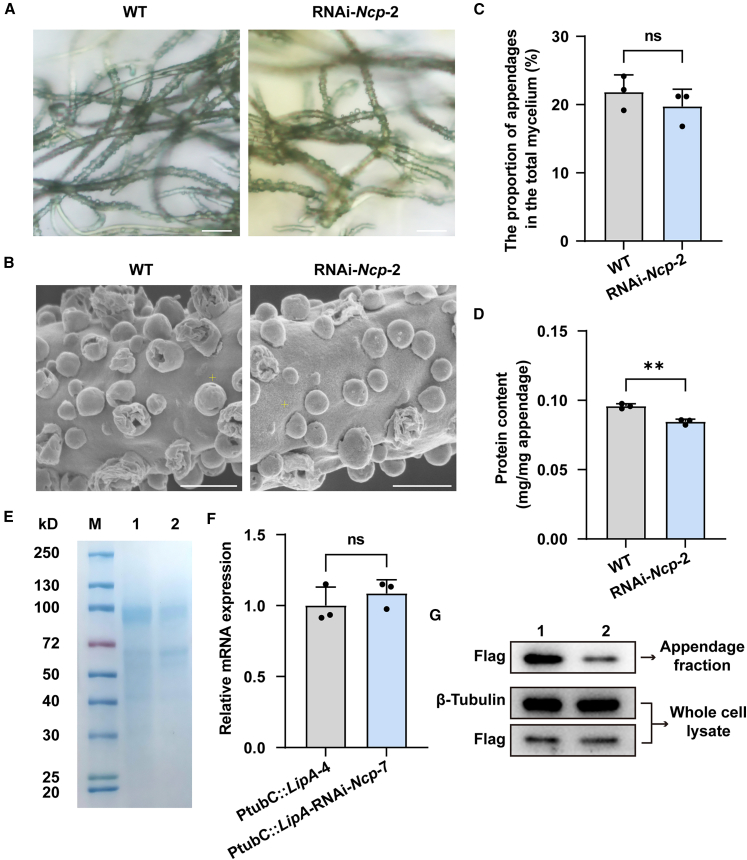
Ncp contributes to protein loading into appendages without affecting appendage formation (A) DIC microscopy (40×) comparing appendage-associated hyphal morphology between WT and RNAi-*Ncp*-2 strains. Scale bars, 20 μm. (B) SEM images showing comparable appendage surface architecture in WT and RNAi-*Ncp*-2 strains. Scale bars, 1 μm. (C) Appendage proportion relative to total mycelial dry weight in WT and RNAi-*Ncp*-2 strains. (D) Total protein content of equal amounts of appendages from WT and RNAi-*Ncp*-2 strains measured by BCA assay. (E) SDS-PAGE analysis of proteins extracted from equal amounts of appendages. Lane 1, WT; lane 2, RNAi-*Ncp*-2. (F) qPCR analysis of *LipA* transcript levels in P*tubC*::*LipA*-4 and P*tubC*::*LipA*-RNAi-*Ncp*-7 strains. (G) Immunoblot analysis of LipA in appendage-associated fractions and whole-cell lysates from P*tubC*::*LipA*-4 and P*tubC*::*LipA*-RNAi-*Ncp*-7 strains; lane 1, P*tubC*::*LipA*-4; lane 2, P*tubC*::*LipA*-RNAi-*Ncp*-7. For (C), (D), and (F), data are presented as mean ± SD from *n* = 3 biological replicates. Statistical significance was determined by two-tailed unpaired Student’s *t* test; ∗∗*p* < 0.01; ns, not significant. See also Figure S3 and Table S1.

Among the five NACHT-domain candidates, *Ncp* exhibited the most robust and upregulation and was selected for functional characterization via RNA interference (RNAi). A representative knockdown strain, *RNAi-Ncp-2*, achieved an approximately 80% reduction in *Ncp* transcript levels (Figure S3A). Morphological analyses by differential interference contrast (DIC) microscopy and SEM revealed no detectable differences in hyphal architecture and appendage morphology between the wild-type (WT) and RNAi-*Ncp*-2 strains (Figures 4A and 4B). Consistently, quantitative measurements of appendage biomass showed no significant difference change (Figure 4C), indicating that Ncp is dispensable for the biogenesis of these extracellular structures.

We next assessed whether Ncp influences protein export into the appendages. Comparative analysis of appendage-associated material, isolated from equivalent culture volumes, revealed a significant reduction in total protein content in the RNAi-*Ncp*-2 strain (Figure 4D). SDS-PAGE analysis further suggested differences in protein composition between WT and RNAi-*Ncp*-2 samples, with reduced abundance of several protein bands in the knockdown strain (Figure 4E). These observations suggest that Ncp contributes to the efficiency of protein export into appendage-associated extracellular material.

To determine whether leaderless proteins are among the cargos influenced by Ncp, we introduced the RNAi construct into the strain heterologously expressing signal peptide-less LipA (P*tubC*::*LipA*-4). A representative transformant, P*tubC*::*LipA*-RNAi-*Ncp*-7, with approximately 74% knockdown efficiency, was selected for further analysis (Figure S3B). qPCR and western blot analyses showed that Ncp knockdown did not alter the intracellular abundance of LipA in whole-cell lysates, but significantly reduced the Lip A content in proteins extracted from equivalent amounts of appendage-associated material using the same extraction protocol, suggesting that Ncp influences LipA export at the level of secretion rather than expression (Figures 4F and 4G). Notably, the reduction of extracellular LipA despite its stable intracellular production, logically implies that the protein is retained within the cytosol when Ncp function is compromised.

Collectively, these findings support the conclusion that Ncp is necessary for the efficient export of at least some leaderless proteins, as exemplified by LipA, into appendage-associated structures. This specialized export process appears to be strictly synchronized with the structural maturation of appendages. While the initial transcriptional surge of *Ncp* occurs at 84 h (G3), our analysis of 5-day-old cultures captured the system at its phenotypic climax, where maximum appendage accumulation provides a robust signal for characterizing UcPS dynamics. These results are consistent with a “developmental gating” model, where Ncp-mediated UcPS is coordinated with the final stages of appendage maturation without affecting their initial biogenesis.

### Ncp modulates PCD-associated phenotypes and ionic stress responses

NACHT domains belong to the STAND ATPase family and are known to drive ATP-dependent conformational changes and oligomerization—biochemical properties frequently associated with the regulation of PCD in diverse eukaryotic systems.^22^ We therefore examined whether Ncp knockdown is associated with changes in PCD-related phenotypes in the Ph506 hyphae.

**Figure 5:**
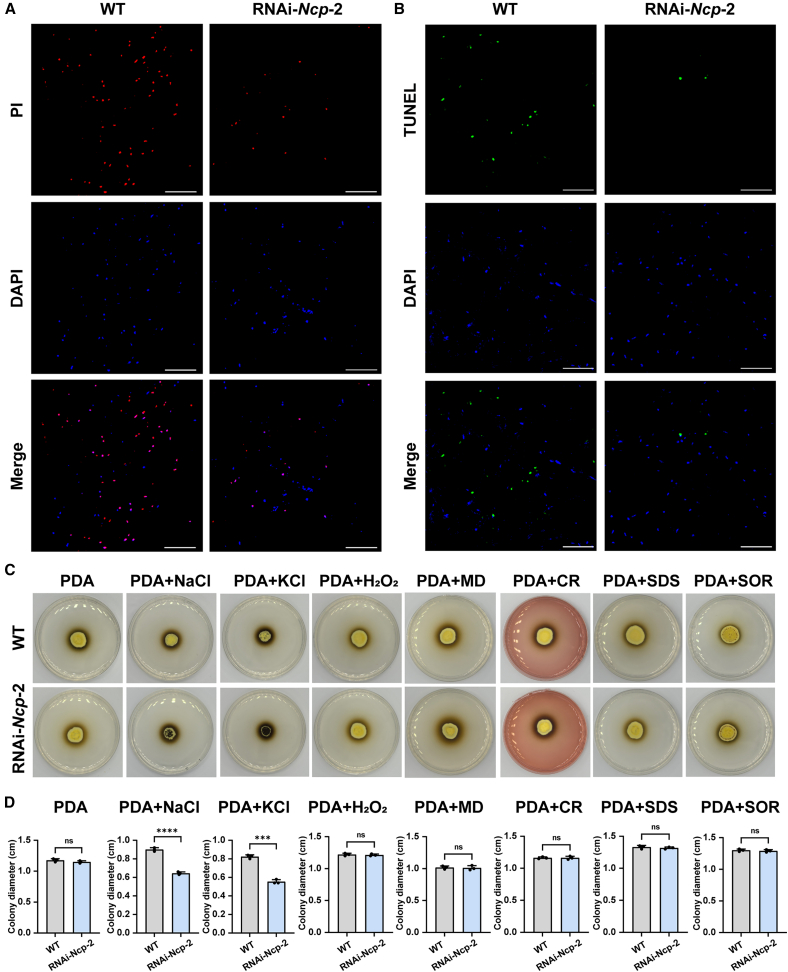
*Ncp* knockdown alters stress responses and cell death-associated phenotypes (A) Fluorescence microscopy of WT and RNAi-*Ncp*-2 hyphae stained with propidium iodide (PI) and DAPI. Scale bars, 25 μm. (B) TUNEL staining of WT and RNAi-*Ncp*-2 hyphae revealing differences in DNA fragmentation. Scale bars, 25 μm. (C) Growth phenotypes of WT and RNAi-*Ncp*-2 strains on various stress-inducing media. (D) Quantification of colony diameters of WT and RNAi*-Ncp*-2 strains under indicated stress conditions. Media included PDA and PDA supplemented with NaCl, KCl, H_2_O_2_, menadione (MD), Congo red (CR), SDS, and sorbitol (SOR). Data are presented as mean ± SD from *n* = 3 biological replicates. Statistical significance was determined by two-tailed unpaired Student’s *t* test comparing WT and *RNAi-Ncp-2* for each condition; ∗∗∗*p* < 0.001, ∗∗∗∗*p* < 0.0001; ns, not significant. See also Figures S4 and S5.

Propidium iodide (PI) staining of 7-day-old hyphae revealed a marked reduction in PI-positive cells in the RNAi-*Ncp*-2 strain relative to WT (Figure 5A). Since PI selectively labels cells with compromised membrane integrity,^44^ this result indicates a decreased proportion of cells undergoing membrane permeabilization upon Ncp knockdown. To further assess PCD-associated processes, we performed TUNEL staining following fixation and permeabilization. Consistent with the PI results, TUNEL-positive nuclei were significantly reduced in the Ncp knockdown strain RNAi-*Ncp*-2 (Figure 5B), indicating reduced DNA fragmentation associated with PCD.^45^ These data suggest that Ncp may be associated with the modulation of PCD-related processes in Ph506, consistent with the established roles of NACHT-domain proteins as molecular switches in cell fate pathways.

Given the observation, we next examined whether Ncp influences broader stress-responsive programs. WT and RNAi-*Ncp*-2 strains were grown under various stress conditions, including ionic (NaCl, KCl),^46^ oxidative (hydrogen peroxide, menadione),^47^ membrane (SDS),^48^ cell wall (Congo red),^49^ and osmotic (sorbitol)^46^ stress. After 7 days, the RNAi-*Ncp*-2 strain exhibited pronounced growth inhibition specifically under high-salt conditions, while no significant differences were observed under other stress categories (Figures 5C and 5D).

This selective hypersensitivity to ionic stress suggests that Ncp is required for robust responses to ion-related challenges rather than maintaining general cellular integrity. Together, these results indicate that Ncp influences both UcPS and stress-related cellular phenotypes, including PCD markers and ionic tolerance. However, whether these processes are mechanistically coupled or represent independent downstream branches of Ncp-mediated signaling remains unresolved and warrants further investigation.

## Discussion

Our data indicate that hyphal appendages in Ph506 function as specialized extracellular compartments^50^ where secreted proteins accumulate. Ultrastructural analyses reveal that appendages are membrane-associated structures external to the cell wall, representing stable domains rather than transient vesicles (Figures 1C and 1D). Whether these cargoes are sequestered within a lumenal space or remain surface-associated remains to be determined and will require further high-resolution spatial mapping. Importantly, these appendages are not exclusive to UcPS, as they accommodate both leaderless and conventionally secreted proteins, supporting their role as general secretion-associated extracellular domains. This spatially restricted secretion mode appears to be an adaptation for surface-associated growth, as appendages are absent in submerged liquid cultures. Because mCherry lacks a signal peptide (Figure S1A), its extracellular localization cannot be explained by canonical ER-Golgi-dependent secretion.^51^^,^^52^ Similarly, the detection of a signal peptide-deleted form of LipA in appendage-associated proteins (Figure 1G) further supports the existence of a non-classical export route for leaderless proteins. The successful export of these two proteins—one a heterologous fluorescent marker and the other an engineered endogenous enzyme—demonstrates that this UcPS-like pathway can accommodate leaderless cargoes with diverse biochemical properties and evolutionary origins.

Consistent with our bioinformatic analysis, the cargoes identified lack canonical N-terminal signals, a hallmark of UcPS (Figure S1). Notably, DeepLoc 2.0 revealed mCherry’s high intrinsic propensity for extracellular localization (0.7843) despite its cytosolic nature in other fungi (Figures S1A and S1C). This “signal-blind but destination-clear” profile, in sharp contrast to the classical export of LipA (Figures S1B and S1D), suggests that cargo selection in Ph506 relies on three-dimensional structural folds or surface charges rather than linear motifs. To further validate this, we performed *de novo* motif discovery using the MEME Suite, which identified no statistically significant conserved motifs among the Ncp-dependent cargoes (all E-values >50; Figures S1E and S1F). Such a mechanism would allow the Ncp-mediated system to selectively export functional enzymes while maintaining cellular integrity. In the case of mCherry and leaderless LipA, their targeted export may be governed by structural and concentration-dependent factors. Specifically, the stable 11-stranded β-barrel scaffold of mCherry provides high structural stability, potentially extending its cytoplasmic “window of opportunity” to be captured by active UcPS machinery.^53^^,^^54^ Furthermore, high cytoplasmic abundance driven by the strong P*tubC* promoter may facilitate export via a “bulk flow” model, where mass action drives stochastic inclusion into active secretory pathways.^16^^,^^55^^,^^56^ Therefore, these cargoes conform to general UcPS substrate characteristics, possessing an intrinsic extracellular propensity despite the lack of sorting signals. In contrast to previously described fungal UcPS routes that often result in diffuse release,^57^^,^^58^ appendage-associated secretion enables spatial confinement—an advantage for symbiotic exchange at defined interfaces.^59^^,^^60^^,^^61^ These observations suggest that Ncp-dependent cargoes are selectively organized into appendage-associated compartments rather than randomly released, providing a structural context for spatially coordinated secretion that integrates UcPS with cellular architecture.

Time-resolved transcriptomic analyses indicate that appendage-associated UcPS is governed by a sustained regulatory program rather than a transient switch. Across G1 to G3, the progressive accumulation of appendages (Figure 2A) mirrors the gradual increase in transcriptional secretory potential observed over this interval. Consistently, a core group of genes remains upregulated throughout this transition, supporting the stabilization of a high-output secretory state. GO enrichment analysis highlights membrane-associated compartments and the cell periphery, aligning with the critical roles of membrane remodeling and vesicular dynamics in UcPS.^6^^,^^7^^,^^62^ Notably, the enrichment of ER-related terms further supports a functional coupling between UcPS and the classical ER-Golgi pathway,^63^ suggesting these systems operate in coordination. Complementing these spatial insights, KEGG analysis reveals the metabolic and stress signaling programs—specifically lipid and carbohydrate dynamics—that drive membrane properties and energy flux.^64^^,^^65^^,^^66^^,^^67^ Collectively, these results indicate that UcPS is a systems-level process integrating metabolic state, membrane organization, and stress adaptation rather than relying on cargo-specific induction.

A notable feature of this transcriptional landscape is the sustained upregulation of NACHT domain-containing genes. As members of the STAND family of P loop NTPases,^19^ these proteins act as ATP-dependent molecular switches capable of integrating multiple signals into coordinated cellular outcomes.^68^ In Ph506, multiple NACHT-encoding genes exhibit expression dynamics that parallel the increase in appendage-associated secretion. Comparative genomic analyses further reveal an apparent expansion of NACHT domain-containing proteins in Ph506 relative to non-symbiotic *Penicillium species* (Figure 3B), suggesting potential regulatory diversification.^69^ While such expansions are noted in stress-adapted lineages, the current limited taxon sampling does not allow for a general association between NACHT expansion and a symbiotic lifestyle or UcPS.^70^

Nevertheless, the potential link between NACHT domain expansion and unconventional secretion across the fungal kingdom remains an open question that warrants broader comparative genomic and functional investigations. Furthermore, the genomic absence of core eukaryotic UcPS components (e.g., ESCRT and GSDMD) combined with the transcriptional stagnation of extant homologs (e.g., Atg8 and Grh1; Table 1; Figure 3C), alongside the functional decoupling of fungal-specific GSDM-like/TMED proteins (Table 2), highlight the prominent role of Ncp in coordinating the Ph506 secretory landscape. Our integrated characterization indicates that while these alternative pathways are either physically missing, structurally incomplete, or transcriptionally inactive during peak secretion, Ncp may function as a primary regulatory switch that bypasses or operates upstream of these canonical systems. In sharp contrast, qPCR confirmed that *Ncp* (10451_g) undergoes a robust 11-fold upregulation from G1 to G3—an expression profile that closely mirrors appendage maturation and justifies its functional characterization (Figure 3A).

Functional characterization suggests that Ncp contributes to efficient UcPS cargo export while being dispensable for appendage biogenesis. Knockdown of Ncp did not affect hyphal morphology or appendage biomass (Figures 4A–4C), indicating that assembly of secretion platforms is genetically separable from regulation of protein loading. In contrast, Ncp depletion significantly reduced total appendage-associated protein levels and altered cargo abundance (Figures 4D and 4E). These patterns suggest that Ncp modulates the efficiency or selectivity of protein incorporation rather than acting as a global driver of secretion, consistent with regulatory nodes that fine-tune energetically costly processes.^71^ The export of the leaderless protein LipA was markedly diminished in Ncp-deficient strains despite unchanged intracellular levels (Figure 4G). According to the principle of mass balance, this reduction logically implies a corresponding retention of cargoes within the cytosol, indicating post-translational regulation at the level of secretion. Given its identity as a NACHT-domain STAND ATPase, Ncp likely functions as a high-level regulatory switch or signaling hub rather than a direct transporter.^72^ While the Ncp-dependency demonstrated for LipA-ΔSP in this study, the co-localization of mCherry suggests a shared, versatile export mechanism. We propose that Ncp functions via a “signal-integrated recruitment” mechanism: upon sensing symbiotic or ionic cues, Ncp may undergo ATP-dependent conformational changes to form a regulatory platform that recruits downstream UcPS-specific translocators or modulates local membrane dynamics. This hypothesis is supported by our GO analysis highlighting membrane-integrated components (Figure 2C). Such a mechanism provides the spatial control necessary to sort soluble proteins, like LipA and mCherry, into burgeoning appendages during “bulk flow” secretion. Thus, Ncp may act as a “gatekeeper” that adjusts UcPS output in response to the developmental state of the appendages. This “developmental gating” ensures that the metabolic cost of secretion is only incurred when extracellular platforms are mature. The temporal lag between the transcriptional surge of Ncp at 84 h (G3) and the phenotypic climax at day 5 is consistent with the time required for translocation and sequestration, justifying our selection of 5-day-old cultures for robust characterization.

The Ncp-dependent pathway represents an alternative UcPS route with significant mechanistic and evolutionary differences from established paradigms. While UcPS is well-characterized in mammals, Ph506 relies on a regulatory framework where many core factors for EV biogenesis (ESCRT) and MAPS (USP19) are entirely absent (Table 1). Crucially, even where homologs exist—such as those involved in autophagy (Atg8), GRASP (Grh1), or fungal-specific GSDM-like/TMED families—their lack of transcriptional response and essential functional motifs (Table 2; Figure 3C) suggests that Ncp-mediated export may not primarily rely on these conventional machineries. However, we cannot exclude the possibility that Ncp acts as an upstream signal activator that converges with highly divergent or post-translationally regulated factors. Beyond molecular differences, the Ncp-mediated system exhibits particular spatial characteristics. Unlike membrane-rupture or direct plasma-membrane translocation, Ncp-dependent cargoes accumulate in discrete extracellular appendages external to an intact, well-defined cell wall (Figure 1D). This structural organization supports a more regulated process rather than a simple lytic or non-selective export model. Similarly, while the mammalian MAPS pathway handles misfolded proteins, the robust secretion of functional enzymes in Ph506 suggests a highly selective recruitment mechanism. Together, these observations support a fungal-specific secretion system that is evolutionarily and mechanistically divergent from the classic UcPS paradigms described in higher eukaryotes.

Beyond its role in modulating UcPS, Ncp is also associated with PCD-related phenotypes in Ph506. Knockdown of Ncp resulted in a reduction of both membrane damage and DNA fragmentation markers (Figures 5A and 5B), consistent with decreased apoptosis-like processes. The parallel decrease of these markers suggests that Ncp may be involved in PCD-related processes, in agreement with the established roles of STAND ATPases in cellular fate regulation.^19^ Importantly, attenuation of PCD-associated markers did not cf. broad stress tolerance. Instead, Ncp-deficient strains exhibited selective hypersensitivity to ionic stress, while responses to oxidative, membrane, cell wall, and osmotic stresses remained unaffected (Figures 5C and 5D). This specificity suggests that Ncp is required for robust responses to ionic challenges rather than maintaining general stress resistance. In filamentous fungi, localized PCD has been proposed to eliminate severely compromised hyphal compartments, thereby preserving network-level function.^73^ We propose that attenuation of this process may allow dysfunctional compartments to persist, which could contribute to impaired adaptation under sustained ionic stress.^74^

Taken together, our findings indicate that Ncp influences UcPS, PCD-associated phenotypes, and ionic stress responses, rather than acting exclusively within a single pathway. While dispensable for appendage biogenesis, Ncp is essential for the efficient export of a subset of leaderless cargoes into these structures; its depletion results in reduced PCD markers and increased sensitivity to ionic stress.^75^ At present, the mechanistic relationships among these processes require additional mechanistic dissection. In filamentous fungi, UcPS provides secretory flexibility when ER-Golgi secretion is constrained,^76^ whereas controlled PCD facilitates the removal of dysfunctional compartments.^21^ Because ionic stress perturbs membrane potential, ion gradients, and cellular energetics, these processes may be influenced under shared physiological conditions.^77^^,^^78^ In the symbiotic lifestyle of Ph506, where long-term nutrient exchange and environmental buffering are critical,^79^ Ncp may function as a context-dependent regulator coordinating secretion and cellular state under persistent stress. Our findings provide a conceptual basis for how UcPS and stress-associated processes are integrated in filamentous fungi, although further work will be required to define the underlying mechanisms.

### Limitations of the study

Although our data are consistent with a role for Ncp in appendage-associated UcPS, the precise molecular mechanism by which Ncp modulates cargo export requires further molecular dissection. In particular, we have not determined whether Ncp directly interacts with potential protein complexes or cargoes, nor have we identified the downstream effectors that execute secretion. As noted by the absence of recognizable homologs for canonical executors, it remains possible that Ncp acts as an upstream activator whose signal converges with highly divergent or post-translationally regulated pore-forming proteins, such as fungal-specific gasdermin-like proteins or TMED family members, which were not captured by our current transcriptomic and domain-based surveys. Furthermore, the absence of genetic complementation or overexpression assays means that potential compensatory or off-target effects, although minimized by our experimental design, cannot be entirely ruled out. In addition, the current study does not capture the full secretome associated with appendages. While we focused on representative proteins to characterize Ncp’s role, future quantitative proteomic analyses will be valuable to define the full spectrum of appendage-associated secreted proteins.

Regarding the functional integration of these pathways, while our results suggest that Ncp influences UcPS, PCD, and ionic stress-related phenotypes, the causal hierarchy among these processes warrants further biochemical investigation. It is currently unclear whether PCD activation serves as a prerequisite for leaderless protein export or whether both processes are parallel responses coordinated by upstream signaling, such as the ionic fluctuations identified in our stress assays. Furthermore, while our findings define a specific regulatory framework in *P. herquei* (Ph506), the current study is limited to a single symbiotic fungal strain under laboratory conditions. Future studies examining the conservation of this system in additional species and *in situ* symbiotic contexts will be valuable to assess the generality and ecological relevance of this regulatory framework.

## Resource availability

### Lead contact

Requests for further information and resources should be directed to and will be fulfilled by the lead contact, Xingzhong Liu (liuxz@nankai.edu.cn).

### Materials availability

This study did not generate new unique reagents.

### Data and code availability

• Data: The genomic and transcriptomic datasets generated during this study have been deposited in the National Genomics Data Center (NGDC) under BioProject PRJCA058011, with the Genome Warehouse assembly accession number GWHHOGM00000000.1 and the Genome Sequence Archive accession number CRA038626. All deposited data are fully and publicly available as of the time of formal submission.
• Code: This paper does not report original code.
• Additional information: All additional resources and materials are available from the lead contact upon reasonable request.

## Acknowledgments

This work was jointly supported by Tianjin Municipal Major Science and Technology Special Projects and Engineering—National Key Laboratory Major Projects (24ZXZSSS00490) and the Startup Fund from the Nankai University to Xingzhong Liu. (030/C029215002). Special thanks to Dr. Manman Qiu for her technical support.

## Author contributions

Conceptualization, L.Y., D.W., and X.L.; methodology, L.Y., D.W., and X.L.; investigation, L.Y., P.Q., X.J.L., P.W., and J.F.; formal analysis, L.Y., W.D., T.L., K.D., and L.Z.; writing – original draft, L.Y. and X.L.; writing – review and editing, L.Y. and X.L.; supervision, D.W. and X.L.; funding acquisition, X.L.

## Declaration of interests

The authors declare no competing interests.

## STAR★Methods

### Key resources table

**Table:** 

REAGENT or RESOURCE	SOURCE	IDENTIFIER
**Antibodies**		

Mouse anti DDDDK-Tag mAb	ABclonal	Cat# AE005; RRID: AB_2770401
*ProteinFind*® Anti-β-Tubulin Mouse Monoclonal Antibody	TransGen Biotech	Cat# HC101-01
*ProteinFind*® Goat Anti-Mouse IgG (H+L), HRP Conjugate	TransGen Biotech	Cat# HS201-01

**Bacterial and virus strains**		

*Escherichia coli* Trans5α	TransGen Biotech	Cat# CD201-01
*Agrobacterium tumefaciens* AGL-1	Coolaber	Cat# CC400

**Chemicals, peptides, and recombinant proteins**		

Calcofluor White	Sigma-Aldrich	Cat# 18909
FM4-64	TargetMol	Cat# T23409
Hygromycin B solution (50 mg/mL)	LEAGENE	Cat# CA0012
G-418 Disulfate	Solarbio	Cat# G8160
Cefotaxime Sodium Salt	Solarbio	Cat# C8240
Kanamycin	Solarbio	Cat# K8020
Phanta Max Super-Fidelity DNA Polymerase	Vazyme	Cat# P505-d1
2 × Rapid Taq Master Mix	Vazyme	Cat# P222-01
Dpn I	NEB	Cat# R0176V
TRIzol LS REAGENT	Invitrogen	Cat# 10296028 C N
Chloroform	Gaofeng	Cat# 0032 A
Isopropanol	Macklin	Cat# I766839
Absolute ethanol	Macklin	Cat# E809056
Methanol	Macklin	Cat# M742213
RNA Extraction Solution (25:24:1)	G-CLONE	Cat# EX0125
5×SDS-PAGE Loading Buffer	Genstar	Cat# E153-05
Pre-stained Protein Marker (10-250 kDa)	Life-iLab	Cat# AP13L062
10×Tris-Glycine Running Buffer	Genstar	Cat# E152-01
10×Western Blot Transfer Buffer (Wet)	Genstar	Cat# E173-01
10×TBST	Genstar	Cat# E175-01
Non-fat milk powder	Solarbio	Cat# LP0033B
2-(N-morpholino)ethanesulfonic acid	BBI	Cat# A420766
Acetosyringone	Macklin	Cat# A800901
D-(+)-Glucose	BBI	Cat# A100188
Glycerol	Macklin	Cat# G810574
K_2_HPO_4_	BBI	Cat# A610447
KH_2_PO_4_	BBI	Cat# A424391
MgSO_4_·7H_2_O	BBI	Cat# A610329
NaCl	BBI	Cat# A610476
(NH_4_)_2_SO_4_	BBI	Cat# A417671
FeSO_4_·7H_2_O	BBI	Cat# A600461
CaCl_2_·H_2_O	BBI	Cat# A610050
KCl	BBI	Cat# A610440
Chelex 100 chelating resin	Macklin	Cat# I902510
PBS Buffer	Solarbio	Cat# P1020
Propidium Iodide	Macklin	Cat# P718302
DAPI	Solarbio	Cat# ID2250
Antifade Mounting Medium	Beyotime	Cat# AC28L532
Triton X-100 (0.1%)	BioSharp	Cat# BL934B
Menadione (MD)	Macklin	Cat# M813096
Congo Red	Solarbio	Cat# IC1000
Hydrogen peroxide solution (H_2_O_2_)	Aladdin	Cat# H112517
Sodium dodecyl sulfate	Solarbio	Cat# S8010
D-Sorbitol	Solarbio	Cat# S8090

**Critical commercial assays**		

BCA Protein Assay Kit	Solarbio	Cat# PC0020
*EasyPure*®Quick Gel Extraction Kit	TransGen Biotech	Cat# EG101-01
*EasyPure*® Plasmid MiniPrep Kit	TransGen Biotech	Cat# EM101-02
HiScript II Q RT SuperMix for qPCR (+gDNA wiper)	Vazyme	Cat# R223-01
2×Universal SYBR Green Fast qPCR Mix	ABclonal	Cat# RK21203
ECL Western Blotting Substrate Kit	BioSharp	Cat# BL520A
One-step 488 TUNEL Apoptosis Detection Kit (Green)	Life-iLab	Cat# AC12L054

**Deposited data**		

*Penicillium herquei* Ph506 Genome	This paper; NGDC	GWHHOGM00000000.1
*Penicillium herquei* Ph506 Transcriptome data	This paper; NGDC	CRA038626

**Experimental models: Organisms/strains**		

*Penicillium herquei* Ph506	This paper; Isolated from *Euops chinensis*	N/A
P*tubC*::*mCherry*-G418	This paper	N/A
P*tubC*::*LipA*	This paper	N/A
RNAi-*Ncp*	This paper	N/A
P*tubC*::*LipA*-RNAi-*Ncp*	This paper	N/A

**Oligonucleotides**		

Primers used in this study	This paper	See Table S1

**Recombinant DNA**		

pFGL-*mCherry*-G418	This paper	N/A
pUC57-*LipA*	This paper	N/A
pFGL-*LipA*-G418	This paper	N/A
pAg-H3-R-*Ncp*	This paper	N/A

**Software and algorithms**		

GraphPad Prism 9GraphPad Software	GraphPad Software	RRID:SCR_002798
OrthoFinder v2.5.1	Emms and Kelly^80^	RRID:SCR_017118
TBtools	Chen et al., 2020	RRID:SCR_018571
HMMER	Eddy, 2011	RRID:SCR_005305
MEGA 11	Tamura et al., 2021	RRID:SCR_000667
OmicShare Cloud Platform	Genescloud	https://www.omicshare.com/tools/
iTOL v6	Letunic and Bork, 2021	RRID:SCR_011754
InterProScan 5	Jones et al.^81^	RRID:SCR_005829
Pfam database	Mistry et al., 2021	RRID:SCR_004726

**Other**		

Immobilon® -E PVDF Membrane	Millipore	Cat# IEVH85R
Micropore filters (0.45 μm)	BIOLAND	Cat# PE33-045

### Experimental model and study participant details

#### Bacterial strains

*Escherichia coli* Trans5α (TransGen Biotech, Beijing, China), with the genotype F^−^ φ80d*lac*ZΔM15 Δ (*lac*ZYA-*arg*F) U169 *end* A1 *rec*A1 *hsd*R17 (r_k_^−^, m_k_^+^) *sup*E44λ- *thi*-1 *gyr*A96 *rel*A1 *pho*A, was utilized for plasmid construction and propagation. *E. coli* strains were cultured in Luria-Bertani (LB) broth or on LB agar plates at 37°C supplemented with kanamycin (100 μg/mL) for selection. *Agrobacterium tumefaciens* strain AGL-1 (Coolaber, Beijing, China), with a *rec*A-deficient C58 chromosomal background harboring nuclear resistance genes for rifampicin and carbenicillin, was employed for *Agrobacterium*-mediated transformation (ATMT) of *Penicillium herquei*. This strain carries a non-self-transmissible, succinamopine-type helper Ti plasmid, pTiBo542ΔT-DNA, which contains an intact vir region to facilitate transfer DNA transfer from binary vectors while its own transfer DNA transfer capability is inactivated. AGL-1 cells were cultured in LB medium at 28°C supplemented with appropriate antibiotic selection (50 μg/mL rifampicin, 100 μg/mL carbenicillin, and 100 μg/mL kanamycin) as required.

#### Fungal strains

The wild-type fungus *Penicillium herquei* strain Ph506 was originally isolated from the leaf-rolling weevil *Euops chinensis*. All wild-type and engineered fungal strains were maintained on Potato Dextrose Agar (PDA) at 28°C. For selection and maintenance of transformants, PDA medium was supplemented with 100 μg/mL G418 or 50 μg/mL hygromycin B as appropriate. During *Agrobacterium*-mediated transformation (ATMT) co-cultivation and subsequent selection phases, 300 μg/mL cefotaxime was routinely added to the medium to completely eliminate residual *Agrobacterium tumefaciens* cells. For long-term preservation, conidia at the mature developmental stage were harvested from 7-day-old cultures and stored in 30% (v/v) sterile glycerol at −80°C. For temporal transcriptomic, secretion, and phenotypic assays, fungal hyphae were characterized and harvested at specific developmental stages, including 36 h (early vegetative growth), 60 h (intermediate development), 84 h (active appendage maturation), and 5 or 7 days post-inoculation (phenotypic climax).

To investigate protein secretion and gene function, several engineered strains with distinct genotypes were constructed using the wild-type Ph506 as the parental background in this study: (1) mCherry-labeled strains (P*tubC*::*mCherry*-G418) expressing cytosolic mCherry under the control of the *tubC* promoter with G418 resistance; (2) signal-peptide-deficient overexpression strains (P*tubC*::*LipA*) carrying the G418 resistance marker to study the non-canonical export of the target enzyme LipA; (3) knockdown strains (RNAi-*Ncp*) harboring an RNA interference cassette targeting the NACHT domain-containing protein gene (*Ncp*) under hygromycin B selection; and (4) dual-functional strains (P*tubC*::*LipA*-RNAi-*Ncp*) combining signal-peptide-less LipA expression and Ncp knockdown, verified under dual antibiotic selection (G418 and hygromycin B). The successful integration of exogenous DNA, selection marker presence, and the expression levels of target genes were validated by genomic PCR, qPCR, and Western blot analysis.

### Method details

#### Scanning and transmission electron microscopy

Fungal mycelia were rapidly frozen in liquid nitrogen and subsequently dehydrated using a freeze dryer. Dried samples were mounted onto aluminum stubs, gently cleaned with compressed air to remove surface debris, and sputter-coated with a 10–15 nm gold layer at a current of 10 mA. Images were acquired using an FEI Apreo S LoVac scanning electron microscope under high-vacuum conditions (∼10^−4^ Pa), with an accelerating voltage of 2.0 kV, a spot size of 7.0, and a working distance of 10 mm. Imaging was performed using the Optiplan objective mode as previously described.^82^

For transmission electron microscopy (TEM), mycelia were fixed in 2.5% (v/v) glutaraldehyde, post-fixed with 1% (w/v) osmium tetroxide, and then dehydrated through a graded ethanol series before being embedded in Epon 812 resin. Ultrathin sections (50–70 nm) were prepared, stained with uranyl acetate and lead citrate, and examined using a Hitachi HT7800 transmission electron microscope operated at 80 kV.

#### Fluorescence staining of cell wall and membranes

CFW staining. For cell wall visualization, mycelia were harvested and washed with PBS. The samples were co-incubated with Calcofluor White (CFW) and 10% (w/v) KOH at room temperature for 5 min in the dark. The staining solution was removed, and the mycelia were washed three times with PBS. CFW fluorescence was observed at 358 nm excitation/461 nm emission.

FM4-64 staining. For plasma membrane staining, mycelia were incubated with FM4-64 at room temperature for 30 min. After washing three times with PBS, the samples were visualized at 535 nm excitation/617 nm emission. All images were captured using a Leica TCS SP5 confocal microscope.

#### Plasmid construction

The mCherry expression vector pFGL-*mcherry*-G418 was maintained in our laboratory. To construct the LipA overexpression plasmid pFGL-*LipA*-G418, the mCherry coding sequence was replaced with a signal peptide-deleted *LipA* gene fused to a C-terminal FLAG tag, using pFGL-*mcherry*-G418 as the backbone. The *LipA* gene (lacking the signal peptide) was PCR-amplified using pUC57-LipA as the template with Phanta Max Super-Fidelity DNA Polymerase (Vazyme) and the primers *LipA*-F/R. RNA interference (RNAi) plasmids pAg-H3-R-*Ncp*, which cf. resistance to Hygromycin B solution, were synthesized by GenScript (China). All primer sequences are listed in Table S1.

#### *Agrobacterium tumefaciens*-mediated transformation and transformant verification

Recombinant plasmids were introduced into *A. tumefaciens* strain AGL-1 by chemical transformation. Positive colonies were verified by colony PCR using 2 × Rapid Taq Master Mix (Vazyme) and cultured on LB agar plates supplemented with Kanamycin. One day prior to transformation, single colonies were inoculated into LB liquid medium containing Kanamycin and cultured at 28 °C with shaking for 20 h.

For preparation of fungal recipient material, wild-type (WT) mycelia were cultured on PDA plates overlaid with sterile cellophane for 5 days at 28 °C until young, actively growing hyphae fully covered the plate. Mycelia were harvested, suspended in sterile water with sterile steel beads, and homogenized to generate a uniform fungal suspension.

*A. tumefaciens* cultures (1.5 mL) were collected by centrifugation, resuspended in 1 mL induction medium supplemented with acetosyringone (IM-AS), and further diluted with 4 mL IM-AS. The induction medium IM-AS was prepared as previously described.^83^ After induction at 28 °C for 6 h with shaking, 1 mL of induced *Agrobacterium* culture was mixed with 1 mL of fungal suspension and spread onto IM-AS agar plates. Plates were incubated at 28 °C for 7 days. Subsequently, the plates were overlaid with PDA medium containing selective fungal antibiotics (Hygromycin B solution or G-418 Disulfate) and the bactericidal antibiotic Cefotaxime Sodium Salt (to eliminate *Agrobacterium*), and incubation continued until the appearance of transformants.^83^ Genomic DNA was extracted from putative transformants using Chelex-100 resin and verified by genomic PCR.^84^

#### Quantitative real-time PCR

Transformants confirmed by genomic PCR were subjected to transcriptional analysis. Total RNA was extracted from fungal mycelia using a phenol-chloroform extraction method. Briefly, samples were homogenized with TRIzol reagent, followed by phase separation through the addition of chloroform. RNA was subsequently precipitated with isopropanol, washed with 75% ethanol, and finally dissolved in RNase-free water according to the manufacturer’s instructions. First-strand cDNA synthesis was performed using the TransScript All-in-One SuperMix kit. Quantitative PCR was carried out on a CFX Connect Real-Time PCR System using gene-specific primers. *β-tubulin* was used as the internal reference gene. Relative gene expression levels were calculated using the 2^-ΔΔCT^ method.^85^

#### Western blot analysis

Protein samples were mixed with 5×SDS-PAGE loading buffer and boiled for 10 min prior to separation by SDS-PAGE.^86^ Proteins were transferred onto PVDF membranes at 250 mA for 40 min. After blocking with 5% (w/v) non-fat milk in 1×TBST for 1 h, the membranes were incubated with primary antibodies, including Mouse Anti-DDDDK-tag (FLAG tag) antibody and Mouse Monoclonal Anti-β-Tubulin antibody (used as an internal control), for 2 h at room temperature. Following three washes with 1×TBST, the membranes were incubated with HRP-conjugated Goat Anti-Mouse IgG (H+L) secondary antibodies for 45 min at room temperature. After three additional washes, protein signals were detected using ECL Western Blotting Substrate Kit and visualized with the ChemiDoc imaging system.^87^

#### Time-resolved transcriptomic analysis

WT conidia were uniformly spread onto PDA medium plates covered with sterile cellophane and incubated at 28°C. Mycelial samples were collected at 36, 60, and 84 h post-inoculation (hpi), with three biological replicates for each time point. RNA sequencing was performed by Biomarker Technologies (Beijing, China). Based on the sequencing results, genes consistently upregulated across all three time points were identified. Subsequently, Gene Ontology (GO) and Kyoto Encyclopedia of Genes and Genomes (KEGG) enrichment analyses were conducted using the OmicShare Cloud platform (https://www.omicshare.com/tools/). GO enrichment was performed based on Fisher’s exact test using a hypergeometric distribution,^88^^,^^89^ and KEGG pathway enrichment was identified by comparing the candidate genes against the whole-genome background.^90^^,^^91^ All *p*-values were adjusted for multiple testing using the Benjamini-Hochberg correction, with a corrected *p*-value (*P*_*adj*_ < 0.05) set as the threshold for significant enrichment.^92^

#### Comparative genomic analysis

OrthoFinder v2.5.1 was employed to analyze the proteomes of 15 genomes using default parameters for the identification of orthologous gene families.^80^ Species tree inference was conducted using the STAG (Species Tree Inference from All Genes) algorithm implemented in OrthoFinder, which generates a species tree based on the most frequently supported bipartitions across all gene trees.^93^ The final tree was midpoint-rooted. Functional annotation of protein domains was performed using InterProScan 5 with Pfam database integration.^81^ The numbers of orthogroups (OGs) and proteins annotated with specific domains were compiled (Table S2). Statistical significance was assessed using one-tailed Student’s t-tests to determine whether the mean number of proteins per orthogroup in Ph506 was significantly greater than the mean across all other species. Phylogenetic trees together with heatmaps and bar charts depicting orthogroup and protein counts were visualized using iTOL.^94^ Furthermore, TBtools was utilized to perform local BLASTP searches (E-value < 1e-(5) against the *P. herquei* Ph506 proteome to further screen and verify specific paralogous or orthologous proteins of interest. The identified protein sequences were then refined and visualized to ensure the accuracy of the comparative genomic findings.

#### Quantification of appendage content

Fungal strains were cultured on PDA plates overlaid with sterile cellophane for 14 days. To collect the extracellular appendages, mycelia were immersed in 50% methanol for 8 min. This extraction condition was optimized based on preliminary experiments demonstrating that the membrane-like appendage structures could be efficiently solubilized by mild organic solvent treatment. The use of 50% methanol facilitates the recovery of appendage-associated extracellular material while maintaining hyphal integrity, thereby minimizing the release of cytosolic contents. Furthermore, immunoblotting confirmed that no obvious protein degradation or loss of epitope detectability occurred under these conditions, supporting the suitability of this protocol for downstream protein analysis.^95^

The eluate was filtered through Micropore filters (0.45 μm) to remove residual hyphae and freeze-dried to obtain appendage dry powder. Mycelial biomass remaining on the cellophane was scraped and dried to constant weight. Appendage content was calculated as the ratio of appendage dry weight to mycelial dry weight. All samples were processed using the same extraction protocol to ensure comparability of appendage-associated measurements between strains.

#### Protein extraction from appendages

Appendage dry powder was repeatedly washed with absolute methanol to remove pigments until the supernatant became colorless. After centrifugation and solvent evaporation, the pellet was resuspended in PBS and mixed with RNA Extraction Solution (25:24:(1) at a 1:1 ratio. The mixture was vortexed and centrifuged at high speed at low temperature. Proteins were collected from the interphase. The extraction procedure was repeated multiple times to maximize protein recovery.^96^^,^^97^

#### Fluorescent staining

PI and DAPI staining. Mycelia were harvested, washed with PBS, and stained with propidium iodide (PI; 5 mg/mL) for 20 min at room temperature in the dark. After washing three times with PBS, samples were stained with DAPI (2 mg/mL) for 5 min, washed, and mounted with antifade mounting medium. Blue fluorescence was observed at 358 nm excitation/461 nm emission, and red fluorescence at 535 nm excitation/617 nm emission.^44^^,^^98^ Images were saved in TIFF format.

TUNEL assay. Mycelia were harvested and processed using the One-step 488 TUNEL Apoptosis Detection Kit (Green) according to the manufacturer’s instructions. Briefly, mycelia were fixed in 4% paraformaldehyde at 4 °C for 30 min, permeabilized with 0.1% Triton X-100 for 20 min, washed with PBS, and incubated with TUNEL reaction mixture at 37 °C for 30 min in the dark. After washing three times with PBS and 0.1% Triton X-100, samples were stained with DAPI (2 mg/mL) for 5 min, and mounted with antifade mounting medium. Blue fluorescence was observed at 358 nm excitation/461 nm emission, and green fluorescence at 490 nm excitation/515 nm emission.^45^^,^^99^ Images were saved in TIFF format.

#### Stress resistance assays

Equal-sized mycelial plugs were inoculated onto the center of 6-cm PDA plates supplemented with different stress agents. Plates were incubated at 28 °C for 7 days. Colony morphology and diameter were recorded. Final concentrations of stress agents were as follows: 0.5 M NaCl, 0.5 M KCl, 0.001% H_2_O_2_, 0.03 mM menadione, 0.01% SDS, 0.2 mg/mL Congo red, and 0.8 M sorbitol.^46^^,^^47^^,^^48^^,^^49^

### Quantification and statistical analysis

All experiments were performed with at least three independent biological replicates. Statistical analysis was conducted using GraphPad Prism 9.0. Data are presented as the mean ± standard deviation (SD). The exact value of *n* and what it represents for each experiment are indicated in the corresponding figure legends. For comparisons between two groups, Student’s *t* test (two-tailed) was employed. For multiple comparisons, one-way ANOVA followed by Tukey’s post hoc test was used. A *p*-value <0.05 was considered statistically significant (∗*p* < 0.05, ∗∗*p* < 0.01, ∗∗∗*p* < 0.001, ∗∗∗∗*p* < 0.0001). No data or subjects were excluded from the analysis.

## Supplemental information

Document S1. Figures S1–S5 and Table S1

Table S2. Comparison of NACHT domain orthologous groups in 15 Penicillium genomes, related to Figure 3
