# Supplementary material for: A NACHT domain-containing protein Ncp is required for appendage-associated unconventional protein secretion in the fungus Penicillium herquei
Source: iScience. 2026 Jun 17;29(7):116464. doi: 10.1016/j.isci.2026.116464 (PMC13311144; doi:10.1016/j.isci.2026.116464)
Supplement: Document S1. Figures S1–S5 and Table S1 [file mmc1.pdf]

iScience, Volume *29*

## Supplemental information

# A NACHT domain-containing protein Ncp is required for appendage-associated unconventional protein secretion in the fungus *Penicillium herquei*

**Luwen Yan, Wei Deng, Penglei Qiu, Tongyao Liu, Kaiwen Deng, Liao Zhang, Xiaojing Liu, Jie Fan, Penglin Wei, Dongsheng Wei, and Xingzhong Liu**

## Supplementary figures and legends

A B

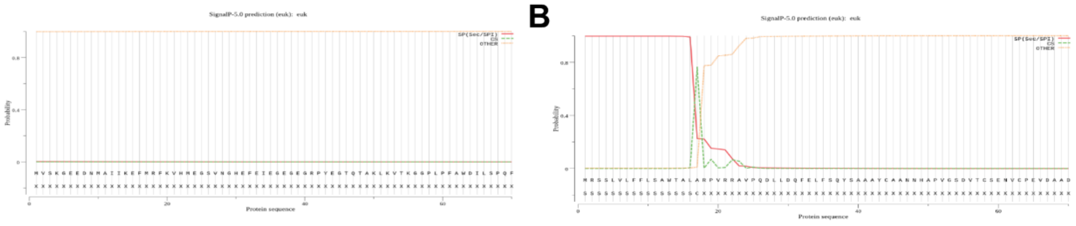


C

**Sequence**

**Predicted localizations:** Cytoplasm, Nucleus, Extracellular

**Predicted signals:**

| Localization | Cytoplasm | Nucleus | Extracellular | Cell membrane | Mitochondrion | Plastid | Endoplasmic reticulum | Lysosome/Vacuole | Golgi apparatus | Peroxisome |
|---|---|---|---|---|---|---|---|---|---|---|
| Probability | 0.5885 | 0.5466 | 0.7843 | 0.0716 | 0.3269 | 0.0502 | 0.1430 | 0.0934 | 0.1630 | 0.0054 |

D

**Sequence**

**Predicted localizations:** Extracellular

**Predicted signals:** Signal peptide

| Localization | Cytoplasm | Nucleus | Extracellular | Cell membrane | Mitochondrion | Plastid | Endoplasmic reticulum | Lysosome/Vacuole | Golgi apparatus | Peroxisome |
|---|---|---|---|---|---|---|---|---|---|---|
| Probability | 0.2110 | 0.0804 | 0.8947 | 0.0358 | 0.0547 | 0.0021 | 0.1402 | 0.3306 | 0.1334 | 0.0032 |

E F

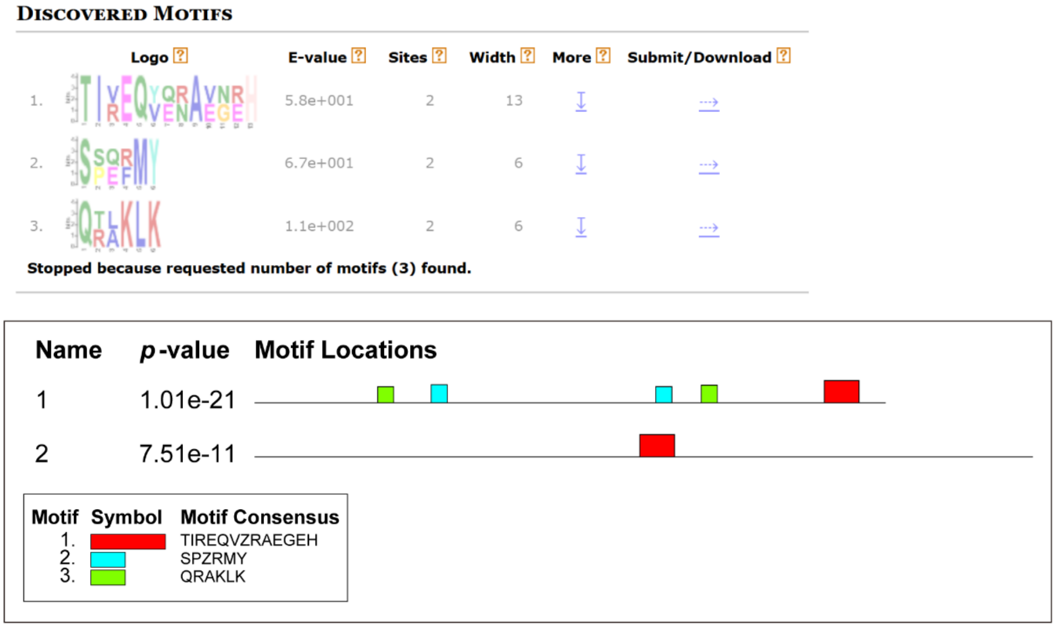


**Figure S1. Bioinformatic characterization of signal peptides, subcellular localization, and motif analysis for mCherry and LipA.** (A–B) Signal peptide prediction by SignalP 6.0 for (A) mCherry and (B) LipA. mCherry lacks a detectable N-terminal signal peptide, while LipA possesses a canonical secretion signal. (C–D) Subcellular localization and sorting signal profile by DeepLoc 2.0 for (C) mCherry and (D) LipA. Despite the absence of a signal peptide, mCherry exhibits a high intrinsic probability for extracellular localization (0.7843). In contrast, LipA shows both a robust signal peptide and a high probability of extracellular localization (0.8947), consistent with its entry into the canonical ER-Golgi pathway. (E–F) *De novo* motif discovery for

mCherry and LipA. (E) Sequence logos and statistical significance (E-values) for the top three motifs identified via pairwise alignment of mCherry and LipA. (F) Positional distribution of the identified motifs across mCherry and LipA sequences. High E-values (> 50) and stochastic localization indicate the absence of a conserved, sequence-based targeting signal.

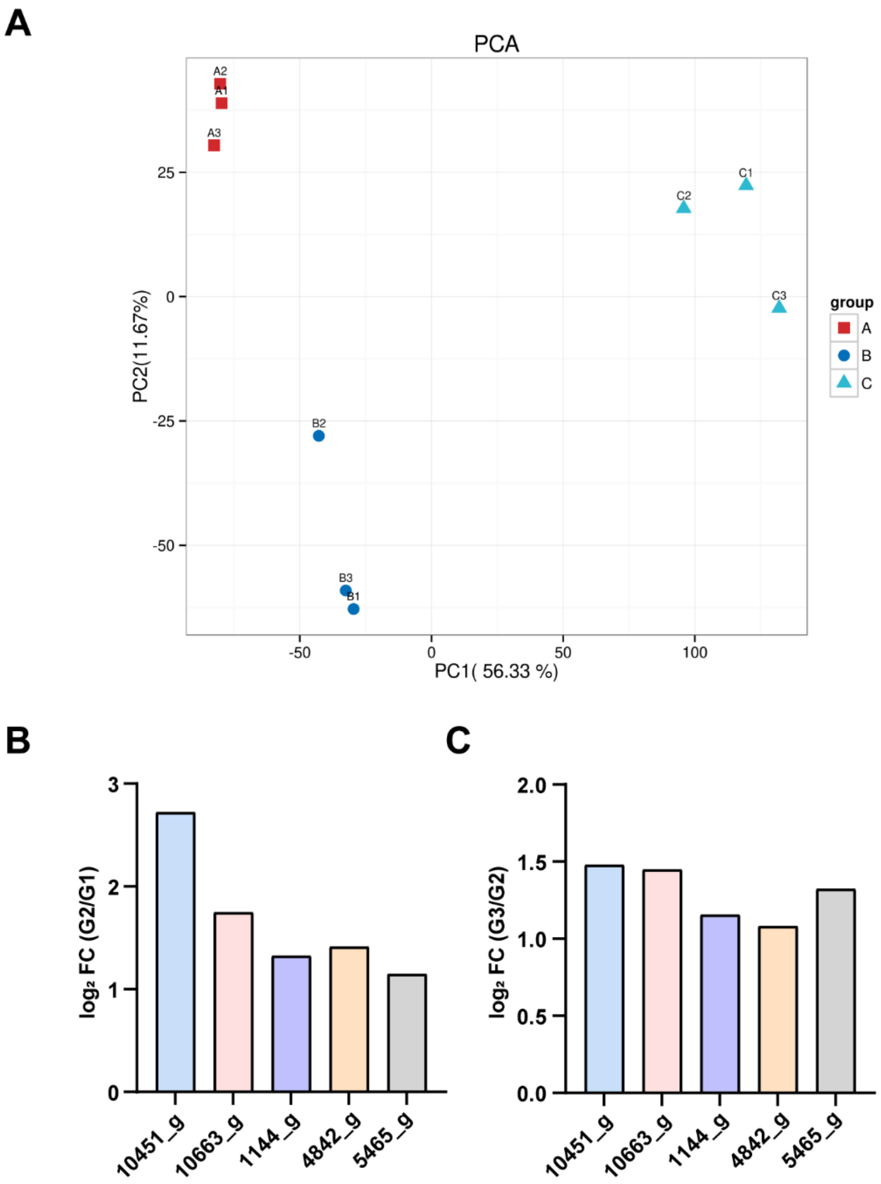


**Figure S2. Temporal transcriptional dynamics of NACHT domain-containing genes during different growth stages.** (A) A, B, and C represent G1, G2, and G3, respectively. (B) Comparative analysis of G1–G2 transcriptional upregulation among NACHT domain-containing genes; the Y-axis represents the $\log_2$ fold change of transcript abundance in the G2 phase relative to the G1 phase. (C) Comparative analysis of G2–G3 transcriptional upregulation among NACHT domain-containing genes; the Y-axis represents the $\log_2$ fold change of transcript abundance in the G3 phase relative to the G2 phase.

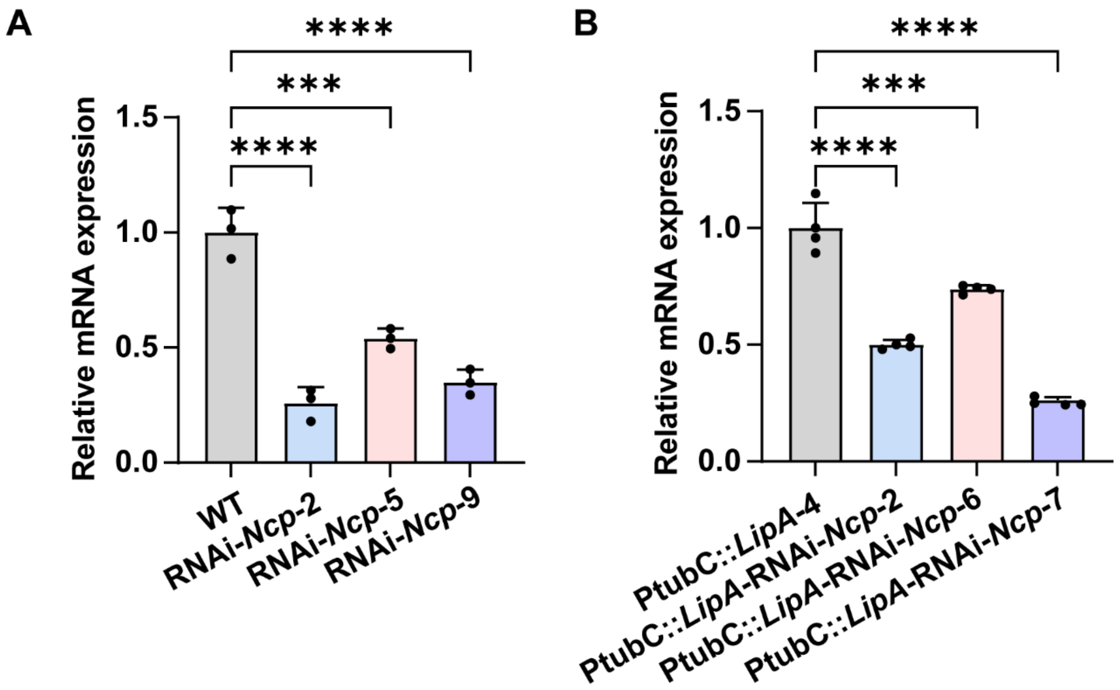


**Figure S3. qPCR validation of Ncp knockdown efficiency in strains used for UcPS assays.** (A) qPCR analysis of *Ncp* transcript levels in RNAi-*Ncp* transformants. (B) qPCR analysis of *Ncp* transcript levels in P*tubC*::*LipA*-RNAi-*Ncp* transformants. Data are presented as mean ± SD from $n = 3$ biological replicates. Statistical significance was determined by one-way ANOVA followed by Tukey's multiple comparisons test; ***$p < 0.001$, ****$p < 0.0001$.

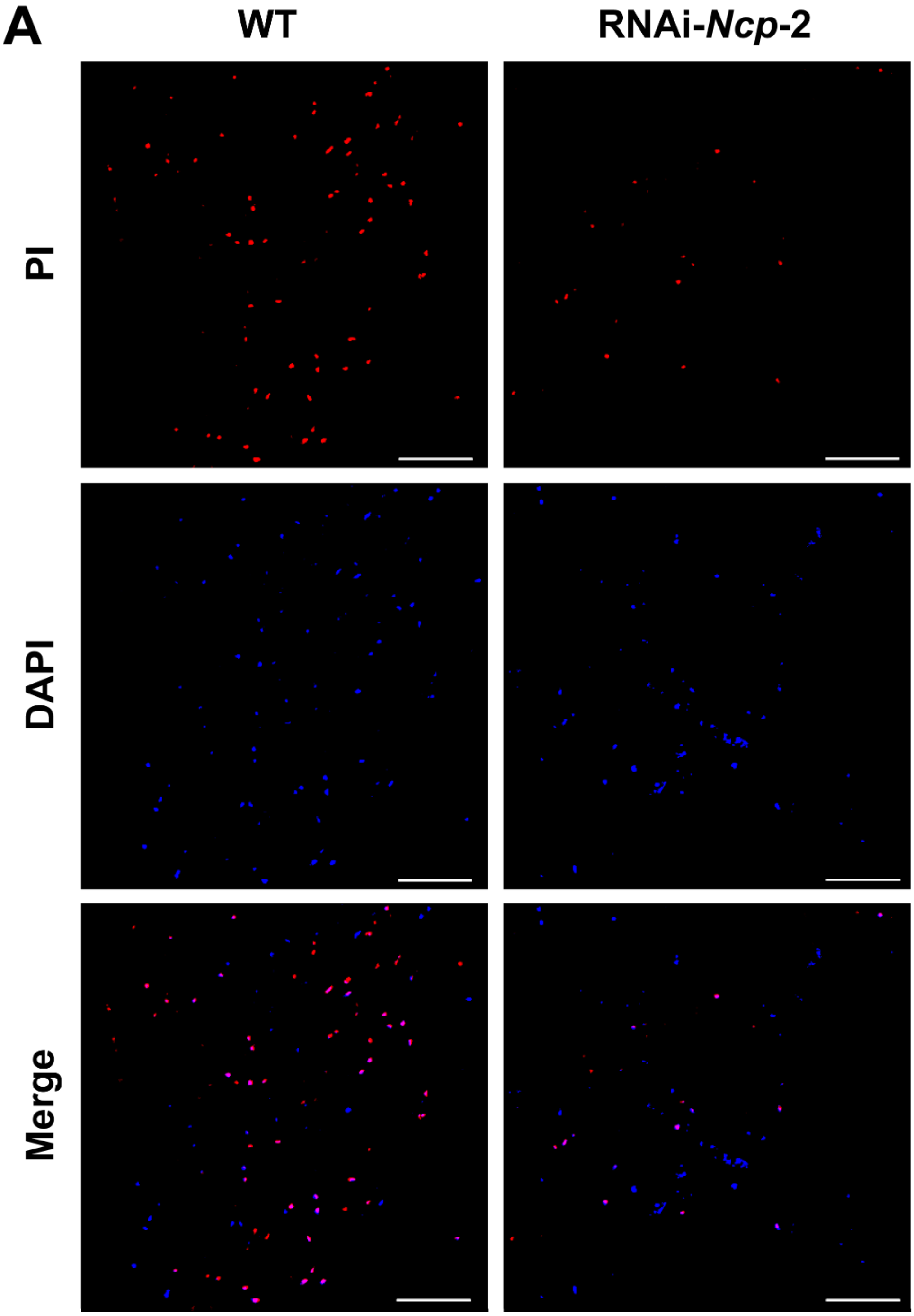


**Figure S4. Magnified fluorescence microscopy of WT and RNAi-*Ncp*-2 hyphae stained with propidium iodide (PI) and DAPI.** Scale bars, 25 µm.

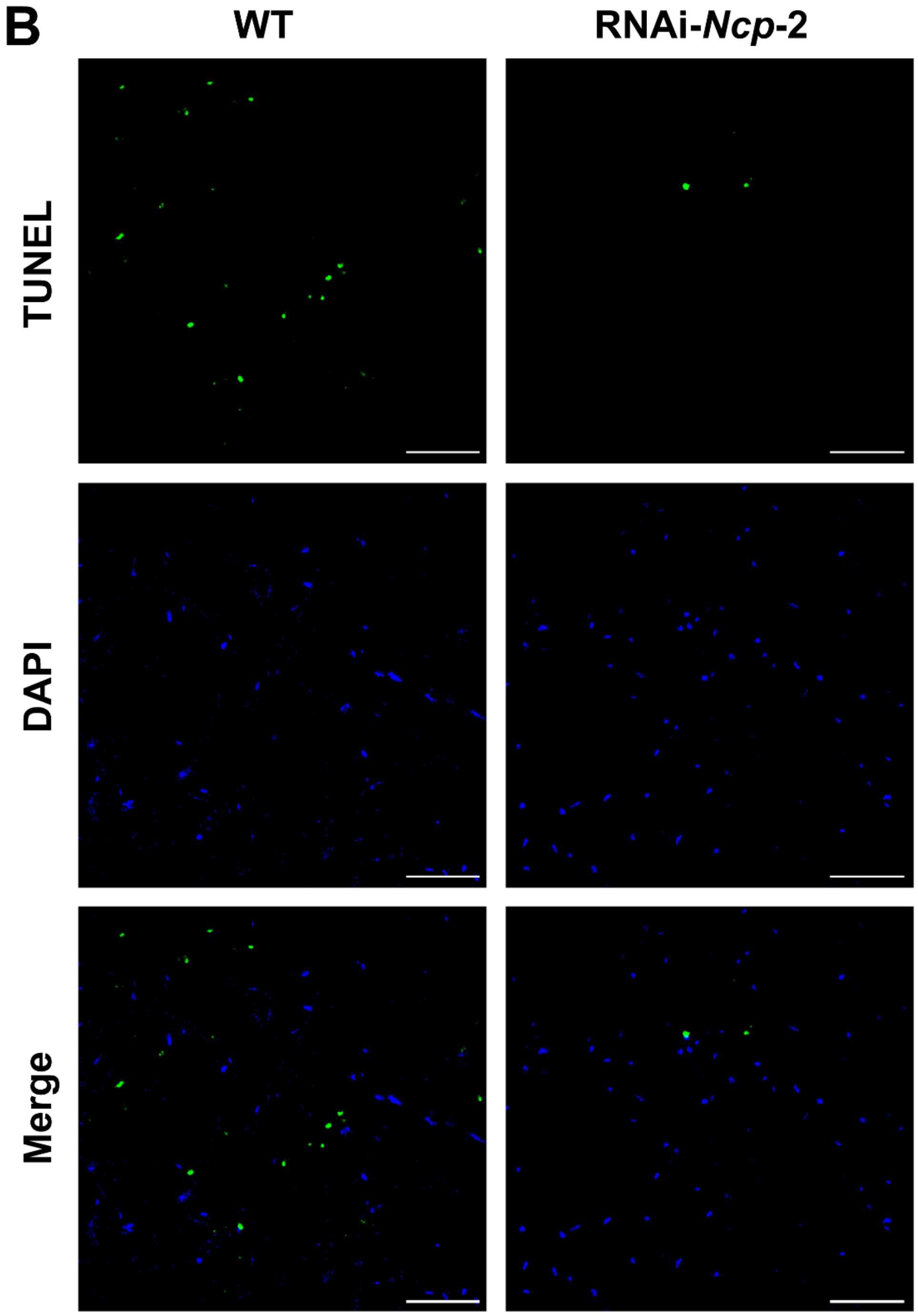


**Figure S5. Magnified fluorescence microscopy of WT and RNAi-*Ncp*-2 hyphae revealing differences in DNA fragmentation by TUNEL assay.** Scale bars, 25 µm.

**Table S1 Primers used in this study.**

| Name | Sequence (5′–3′) | Purpose |
|---|---|---|
| *LipA*-F | CAGCTCACTTATCGTCGTCATCCTTGTA ATCATCACACTCTGAAATGGGACCG | Amplification and Validation of *LipA* |
| *LipA*-R | GTCCCGTTTACGACATTTTAACAAATC GTCAATATGCGGCCTGTTCGACGAGC | |
| *mCherry*-F | CTTGTACAGCTCGTCCATGC | Validation of *mCherry* |
| *mCherry*-R | GAGCAAGGGCGAGGAGGATA | |
| *Hyg*-F | CGAAGAATCTCGTGCTTTCAGC | Validation of *Hyg* |
| *Hyg*-R | GTCAAGACCAATGCGGAGC | |
| q*LipA*-F | AGTACTCCGGGCTAGAGTGG | qPCR validation of *LipA* |
| q*LipA*-R | CGTCGGTAACAGGGCATTTG | |
| q*Ncp*-F | TGACGTGGTCAACACCCTTG | qPCR validation of *Ncp* |
| q*Ncp*-R | CTTGAAGGAATACGCTGATGTCG | |
| q10663-F | GTCATAGCATTTGCCCTGGATAC | qPCR validation of 10663_g |
| q10663-R | ATCTTCCGCGTAATCCATGC | |
| q1144-F | ATGTCACCAAGCCGTATGTCT | qPCR validation of 11144_g |
| q1144-R | CCCAACCACTACACCCTCG | |
| q4842-F | GTCCTCTCTTCCGCAGTGAT | qPCR validation of 4842_g |
| q4842-R | GCATGTTCTTTGCACATCGAG | |
| q5464-F | TTTGCAAAGCGCGACAATGA | qPCR validation of 5464_g |
| q5464-R | CCTCAGAACGTCTAGCGTTCT | |
| q*tubulin*-F | CTGGTAACAACTGGGCTAAGG | qPCR validation of *β-Tubulin* |
| q*tubulin*-R | ACGGAGAAGGTAGCCATCATA | |
